# Progress in Medicine

**Published:** 1901-04-13

**Authors:** 


### Progress in Medicine.

**Typhoid Fever.**—The John Hopkins Hospital Reports, vol. viii., No. 3-8, are devoted exclusively to the consideration of enteric fever in its surgical, medical, and bacteriological aspects. Finney and Cushing contribute articles on the surgical treatment of perforation. The former states that there is no pathognomenic sign of perforation, but that it should be suspected if there be an attack of severe, continued abdominal pain with nausea and vomiting, and an increase in the number of white blood corpuscles. If there be any doubt as to the diagnosis an exploratory incision should be made, and for this purpose, and for the subsequent operative procedures, he recommends cocaine anæsthesia. The necessity for early operation is also urged by Cushing. In his opinion it is possible by a careful blood count to diagnose a pre-perforative stage, and it is in this stage that operation will be most successful. Whilst in uncomplicated enteric fever the number of leucocytes per cubic millimetre is at or below the normal, the advent of an inflammatory complication is signalised by a rise in the leucocyte count. In the circumscribed, slowly-forming peritonitis, after perforation this excess of leucocytes in the peripheral circulation is maintained, but if general peritonitis ensue, the count drops, consequent upon the great outpouring of leucocytes into the peritoneal cavity. A rise in the number of leucocytes should then, if accompanied by abdominal symptoms, lead to the consideration of operative measures. Flexner describes some unusual forms of infection with typhoid bacillus with especial reference to typhoid septicæmia. In this condition the symptoms are those of a general septicæmia with few abdominal symptoms. Few or no typhoidal lesions are to be recognised in the viscera after death, and the diagnosis rests on the presence of Widal's reaction, and the recovery of the specific bacillus from the organs or general circulation. The writer has collected some forty cases of this nature. Cases of multiple infection in enteric fever are not common. Two are recorded: in one the B. Coli, B. pyocyaneus and B. Proteus were isolated, in the other B. Coli, B. Proteus and streptococci. Flexner also records a case of peritonitis in which the only organisms present were the B. typhosus and streptococci. He mentions, too, some interesting cases of ulceration of the gall-bladder, larynx and œsophagus occurring in typhoid fever. This last subject is dealt with more extensively by Mitchell in an article on the œsophageal complications of typhoid fever. He describes the following pathological conditions:—Catarrhal inflammation of the œsophagus, in some cases this takes on a follicular type, the glands appear swollen, and little plugs consisting of mucus and cells can be squeezed from them; diphtheritic membrane forming on the œsophagus; gangrene of the œsophagus; ulceration of the œsophagus, the ulcers being of the typhoid type, lying horizontally, and in the upper part of the tube. Stricture of the œsophagus may follow the cicatrisation of the ulcers, three cases are given in detail. Spasm of the œsophagus may cause difficulty of swallowing in enteric fever, or the dysphagia may be due to any of the above-mentioned causes. Hæmatemesis in this disease is more commonly due to an ulcer in the stomach or to hæmorrhagic infiltration of the stomach walls. The organism most frequently associated with these œsophageal lesions is the streptococcus. In no case has the Erberth-Gaffky bacillus been isolated.

Cases of hæmorrhagic typhoid fever are not uncommon. The most frequent site of the hæmorrhage is the gums, and large quantities of blood may be lost leading to profound anæmia. The skin is nearly always implicated, the petechiæ being most profuse on the trunk and arms, especially on the posterior surfaces. There may in addition be epistaxis, hæmaturia, and extensive sanious effusion into the pleuræ, peritoneum, and meninges. The coagulation time is prolonged. Hæmorrhagic typhoid may be due to a peculiar epidemic type of the disease, and is also said to be common in persons of the hæmorrhagic diathesis. Its direct causation is a profound alteration of the blood. Two-thirds of the cases die. Details of a case are given by Hamburger.

Puerperal infection by the bacillus typhosus is extremely rare. Kühnau describes one such case in which he obtained cultures of the typhoid bacillus from the blood and found the same organism together with strepto- and staphylococci in the uterus. In the case recorded by

Dobbin the typhoid bacillus was found in the uterine lochia associated with streptococci. Pyæmic abscesses containing streptococci formed in various parts of the body, but the patient made a good recovery. Camac summarises his conclusions with regard to the gall-bladder complications of typhoid fever as follows :—Typhoid bacilli enter the gall-bladder from the gastro-intestinal tract by means of the portal system. This can only occur when the bactericidal action of the liver cells has been paralysed by the typhoid toxins. The bacilli may escape from the gall-bladder and reinfect the system. Thus early feeding of enteric cases by leading to an outpouring of the bile may excite relapses. If the bile duct becomes plugged by mucus or a stone, the bacilli may set up cholecystitis, ulceration and perforation; this may even occur after the lapse of several years. That typhoid bacilli may lead to the formation of gall-stones is not proved. The signs of cholecystitis in typhoid fever are pain and tenderness over the gall-bladder, the presence of a tumour, and occasionally jaundice. With regard to treatment, counter-irritation and aspiration are unsatisfactory; the gall-bladder when distended should be opened and drained. The mortality from operation following spontaneous rupture is very high; other liver complications are dealt with by Osler. Small focal necroses are found in the liver at post-mortem examination in nearly all cases. They are detectable by the naked eye and may lead to the formation of fibrous nodules. Jaundice is either due to catarrh, to toxic poisoning, abscess, gall-stones, or cholangitis. The same writer also gives details of four cases of hemiplegia following or occurring during typhoid fever. In the first there were severe convulsions on the ninth day, speedily terminating fatally. Post-mortem examination showed thrombosis of the ascending parietal and parieto-temporal branches of the middle cerebral artery. In a second fatal case the onset of the hemiplegia in the third week was gradual and accompanied by Cheyne-Stokes breathing and delirium. The lesion was thrombotic softening of the right internal capsule. Of the other two cases, one was ushered in with convulsions in the tenth week, and the other had a gradual onset at the end of the second week. Both recovered with permanent paralytic symptoms.

Cystitis due to presence of the typhoid bacillus is a well recognised condition, but the case reported by Young is remarkable from the fact that it was of seven years' duration. There were never any irritative symptoms, and the patient's attention was drawn to the condition solely by the sediment in the urine. During the progress of the case there was superadded an attack of gonorrhœa and a gonococcus infection of the bladder. This was far more resistant to remedial treatment, including urotropin, than was the typhoid infection. The disinfection of a typhoid urine may, according to Gwyn, be immediately effected by the addition of half of its volume of 1 in 20 carbolic acid solution, or one-fifth of its volume of 1 in 1,000 perchloride of mercury solution, or three-tenths of its volume of ten per cent. formalin solution. A detailed account of the blood in complicated and uncomplicated cases of enteric fever is furnished by Thayer. In the febrile stage there is a progressive diminution of both blood corpuscles and hæmoglobin. The average loss of red cells is about one million per cubic millimetre. The white cells too are diminished throughout the attack. Cold baths, inflammatory complications (abcesses, phlebitis, peritonitis, pleurisy, pneumonia, periostitis, cystitis) and hæmorrhages may increase the number of white cells and an increase to ten thousand to the cubic centimetre should lead to a suspicion of one or other of these. The percentage of polymorphonuclear leucocytes and eosinophils is not increased in enteric fever, the only cells showing a relative increase being the medium-sized lymphocytes. The leucocytosis which accompanies perforation of the bowel may disappear as suppurative peritonitis becomes general, or may be entirely absent in very malignant cases. A pre-perforative leucocytosis due to local peritonitis about deep ulcers may occur.

